# Geostatistical Microscale Study of Magnetic Susceptibility in Soil Profile and Magnetic Indicators of Potential Soil Pollution

**DOI:** 10.1007/s11270-015-2395-5

**Published:** 2015-04-14

**Authors:** Jarosław Zawadzki, Piotr Fabijańczyk, Tadeusz Magiera, Marzena Rachwał

**Affiliations:** Environmental Engineering Faculty, Warsaw University of Technology, Nowowiejska 20, 00-653 Warsaw, Poland; Department of Land Protection, Opole University, Oleska 22, 45-052 Opole, Poland; Institute of Environmental Engineering, Polish Academy of Sciences, Sklodowskiej-Curie 34, 41-819 Zabrze, Poland

**Keywords:** Field magnetometry, Magnetic indicators, Soil pollution, Soil magnetic susceptibility, Spatial correlation

## Abstract

Directional variograms, along the soil profile, can be useful and precise tool that can be used to increase the precision of the assessment of soil pollution. The detail analysis of spatial variability in the soil profile can be also an important part of the standardization of soil magnetometry as a screening method for an assessment of soil pollution related to the dust deposition. The goal of this study was to investigate the correlation between basic parameters of spatial correlations of magnetic susceptibility in the soil profile, such as a range of correlation and a sill, and selected magnetometric indicators of soil pollution. Magnetic indicators were an area under the curve of magnetic susceptibility versus a depth in the soil profile, values of magnetic susceptibility at depths ranging from 1 to 10 cm, and maximum and background values of magnetic susceptibility in the soil profile. These indicators were previously analyzed in the literature.

The results showed that a range of correlation of magnetic susceptibility was significantly correlated with magnetic susceptibility measured at depths 1, 2, and 3 cm. It suggests that a range of correlation is a good measure of pollutants’ dispersion in the soil profile. The sill of the variogram of magnetic susceptibility was found to be significantly correlated with the area under the curve of plot of magnetic susceptibility that is related to the soil pollution. In consequence, the parameters of microscale spatial variability of magnetic susceptibility in s soil profile are important measures that take into consideration the spatial aspect of s soil pollution.

## Introduction

Field magnetometry is a cost-effective method that enables simple and quick measurements of soil contamination caused by industrial dusts (Strzyszcz et al. 1996; Kapička and Petrovský 1997; Petrovský et al. 2000; Boyko et al. 2004; Magiera et al. 2007). Numerous studies confirmed significant correlation between magnetic susceptibility as an indicator of industrial dust concentration in the uppermost soil horizons and a concentration of heavy metals (Georgeaud et al. 1997; Schibler et al. 2002; Desenfant et al. 2004; Spiteri et al. 2005; Wang and Qin 2005). Additionally, numerous complementary techniques are still developed that intend to integrate magnetometric measurements with the geochemical ones and even with remotely sensed data (D’Emilio et al. 2012).

Studies of spatial correlations of soil magnetic susceptibility measured on the soil surface (Magiera and Zawadzki 2006; Zawadzki and Fabijańczyk 2007) and in the soil profile (Zawadzki et al. 2012) allow not only for better understanding of the vertical distribution of soil pollution but it can also make it possible to integrate different types of measurements by geostatistical methods, like cokriging.

Soils of forested areas, which are usually used as study sites in field magnetometry, are often characterized by an increased concentration of technogenic magnetite. Moreover, the dissolution of the technogenic magnetite has a significant influence on the accumulation and distribution of heavy metals in the soil profile (Vodyanitskii 2013). Studies suggest also that the distribution of heavy metals in soil profile depends strongly on the composition of the soil, especially the presence of the carriers of heavy metals (Vodyanitskii 2014). In a consequence, the determination of the soil pollution should be accompanied by the analyses of soil profiles (Vodyanitskii and Yakovlev 2011).

The SM-400 device (Petrovský et al. 2004) enables to measure the magnetic susceptibility in the soil profile, and the result is a distribution of the magnetic susceptibility in the soil profile that has numerous properties correlated with the natural and anthropogenic magnetic particle distribution. Plots of magnetic susceptibility were successfully used to differentiate between the magnetic enhancement that was caused by anthropogenic pollution or by natural lithogenic origins (Magiera et al. 2006; Fialová et al. 2006). The distribution of magnetic susceptibility in the soil profile was also used to determine several magnetometric indicators of soil pollution (Zawadzki et al. 2008). The maximum value of magnetic susceptibility was found to be strongly correlated with the highest concentration of heavy metals in the soil profile and was also observed at the same depth in the soil profile (Spiteri et al. 2005). The area under the curve of magnetic susceptibility versus the depth in the soil profile was also found to be correlated with the concentration of heavy metals (Hanesch and Scholger 2002; Spiteri et al. 2005; Blaha et al. 2008; Zawadzki et al. 2008).

The goal of this study was to investigate the relation between parameters of spatial correlation of magnetic susceptibility in the soil profile and selected magnetometric indicators of soil pollution. Such investigation was not carried out so far, and what is very important in this type of study is crucial for further development of the soil magnetometry as a screening technique for soil pollution study. It is also necessary for the standardization of this method and the development of the integrated measurement of soil magnetic susceptibility. In order to achieve this, series of SM-400 measurements were carried out in the forested area located in the Upper Silesian Industrial Area. The readings of magnetic susceptibility were made with the interval of about 1 mm, so it enabled to investigate the microscale spatial variability. Next, variogram analysis was performed, and parameters characterizing the spatial variability of magnetic susceptibility in the soil profile were determined. After that, the correlation between these parameters and selected magnetometric indicators of soil pollution was analyzed.

## Materials and Methods

### Study Area

The study area was located in Upper Silesian Industrial Region, placed in Silesian Voivodeship in southern Poland. The study area covered about 5 km^2^ of forested area neighboring the old, post-mining area that was intensively used for extraction of silver, zinc, and other minerals since tenth century. The geological bedrock of the study area was composed of Triassic rock complex including limestones, marlstones, and ore-bearing dolomites rich in Fe, Pb, and Zn. Metals in dolomites occurred mostly in sulfides of Pb and Zn, sulfides of Fe, and also carbonates of Pb and Zn (Cabała et al. 2004). The Triassic formation was covered only with a thin layer of eolian sands. Due to the past intense ore exploration, the anthropogenic pressure was, and still remains, significant.

**Fig. 1:**
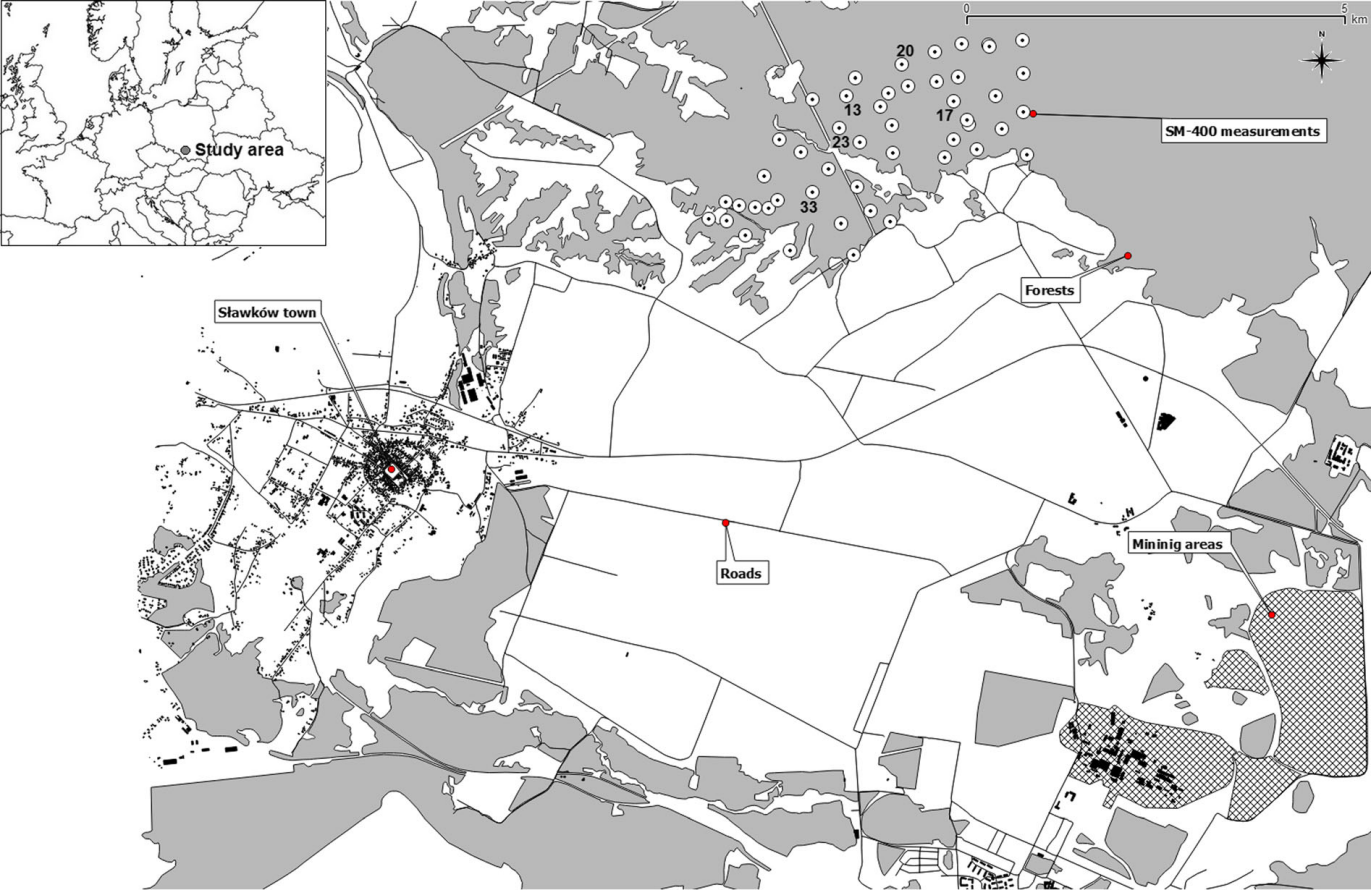
The vicinity of the study area and the location of sample points where magnetic susceptibility was measured with SM-400 in the soil profile; for *labeled points*, the distributions of magnetic susceptibility in the soil profile were presented in the Fig. 2

At present, the neighborhood of the study area is mostly occupied by arable lands with moderately dense net of paved roads and sparse residential buildings. Only mines “Bolesław” and “Olkusz-Pomorzany” are still active (Fig. 1).

### Measurements of Soil Magnetic Susceptibility

The measurements of soil magnetic susceptibility in soil profile were performed with specially designed device SM-400 (Petrovský et al. 2004). The main part of the SM-400 is a plastic tube with the magnetic sensor inside that is being moved upwards and downwards during the measurement. Before the measurement, the 30-cm-deep drilling was made using HUMAX SH 300 sampler. Next, the tube of SM-400 was inserted into prepared hole, and the measurement of soil magnetic susceptibility was performed twice, so as to reduce the influence of the temperature on the inductivity of the probe. For further analyses, the average from these two measurements was used. The reading interval of magnetic susceptibility was equal to about 1 mm, starting at the soil surface and finishing at the depth of about 20 cm. Values of volume specific magnetic susceptibility (κ) were dimensionless, expressed in 10^−5^ SI units. In total, 49 measurements of magnetic susceptibility in the soil profile were made.

### Variogram Calculation Methods

The parameters of microvariability of soil magnetic susceptibility in the soil profile were investigated using semivariance (Goovaerts 1997) and classic variograms. The variograms were calculated as directional variograms, with the direction along the soil profile. After the experimental variograms were calculated, they were modeled with the same model, and the range of correlation, nugget effect and the sill were noted for further analyses.

**Fig. 2:**
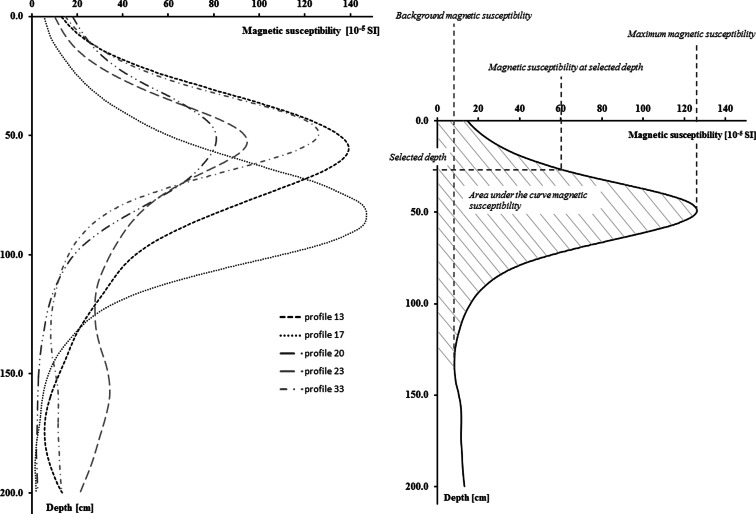
Plots of soil magnetic susceptibility in the soil profile measured with SM-400 at points that were labeled in the Fig. 1 (*left plot*); magnetic indicators that were calculated and used in analyses (*right plot*)

The distribution of κ values in the soil profile is usually characterized by specific shape. Starting from the soil surface, magnetic susceptibility increases rapidly with the depth, achieves maximum value at the depth of about 3 to 6 cm, and decreases at deeper parts of soil profile (Fig. 2). In a consequence, it was necessary to reduce the trend from the magnetic susceptibility distribution in the soil profile. The trend was modeled using polynomials and then subtracted from the raw data. After that, all analyses of spatial variability were carried out on the residual values of magnetic susceptibility. Directional variograms were calculated separately for each measurement point, so in the result, the set of 49 variograms were achieved.

### Calculation of Magnetic Indicators

Magnetic indicator was a value or measure that was correlated with soil pollution with heavy metals and was calculated using plots of magnetic susceptibility in soil profile. All magnetometric indicators used in this study were previously analyzed (Zawadzki et al. 2008), and their significant correlation with soil pollution with heavy metals was confirmed. Magnetometric indicators were presented in the Fig. 2.

First set of magnetometric indicators included the values of magnetic susceptibility in the soil profile at the depths ranging from 1 to 10 cm that were calculated using SM-400 plots. Additionally, also maximum, background and average magnetic susceptibility was calculated.

The area under the curve of magnetic susceptibility versus the depth in the soil profile was calculated using script that was written specially for this purpose in MATLAB. The area was calculated to the depth of 6, 8, 10 cm, and to the depth of background value of magnetic susceptibility in the soil profile (Fig. 2). The resulted areas were expressed in millimeter 10^−5^ SI.

## Results and Discussion

In the study area, distributions of κ values in the soil profile were characterized by a shape with one well-visible peak at the depth of several centimeters (Fig. 1). Distributions of magnetic susceptibility in the soil profile were previously analyzed by Magiera et al. (2006), and the distribution observed in this study was characteristic for forested areas with high anthropogenic pressure and was marked by Magiera et al. (2006) as a type A1.

Maximum values of magnetic susceptibility observed in 49 measurement points were located between 4th and 6th centimeter in the soil profile. The average depth of maximum magnetic susceptibility was equal to 5.9 cm (Table 1). Maximum κ values in the soil profile were exceeding 100 [×10^−5^ SI units] that was indicating significant soil pollution.

**Table 1:** Descriptive statistics of magnetic indicators, a range of correlation and a sill of vertical variograms

Magnetic susceptibility		Mean	Median	Minimum	Maximum	Std dev.	Skewness
At depth of 0 cm	[×10^−5^ SI]	18.27	16.13	1.55	55.03	12.82	1.12
At depth of 1 cm		32.59	25.43	1.38	103.12	24.19	1.23
At depth of 2 cm		50.61	43.45	2.73	146.06	34.92	1.05
At depth of 3 cm		68.60	61.83	4.83	160.27	38.82	0.37
At depth of 4 cm		84.33	84.53	8.48	179.71	40.99	−0.01
At depth of 5 cm		93.53	93.82	13.61	217.54	43.71	0.23
At depth of 6 cm		93.16	81.67	16.50	229.28	47.07	0.57
At depth of 7 cm		86.07	76.68	10.59	210.25	50.39	0.77
At depth of 8 cm		74.09	57.83	7.95	189.97	48.03	0.73
At depth of 9 cm		60.30	51.65	3.40	170.95	42.18	0.67
At depth of 10 cm		47.71	35.29	0.32	136.16	37.68	0.84
Maximum		127.38	130.50	64.10	229.50	36.25	0.35
Background		0.08	0.04	0.00	0.57	0.12	2.59
Depth of maximum	[mm]	59.0	50.0	30.0	110.0	20.5	0.85
Depth of background	[cm]	13.95	14.00	7.00	19.00	2.84	−0.10
Area—to background value	[mm 10^−5^ SI]	78.41	76.42	22.05	157.93	28.31	0.47
Area—to the depth of 6 cm		38.41	39.50	4.10	77.80	18.03	−0.02
Area—to the depth of 8 cm		55.54	57.50	11.00	118.60	22.60	0.17
Area—to the depth of 10 cm		67.74	68.30	22.00	140.70	24.94	0.57
Range of correlation	[mm]	48.38	48.00	27.00	105.00	14.56	1.25
Sill	[×10^−10^ SI]	0.39	0.35	0.01	0.93	0.26	0.74

Average background value of magnetic susceptibility was close to 0 [×10^−5^ SI units] that was mainly resulted from the low, almost diamagnetic, κ value of quartz sand in illuvial horizon of Podzols. On average, the background value of magnetic susceptibility was observed at the depth of about 14 cm (Table 1 and Fig. 2).

All variograms of magnetic susceptibility in the soil profile were characterized by no nugget effect. The lack of the nugget effect resulted from the very dense readings of magnetic susceptibility, about 1 mm, and suggested that the measurements performed the SM-400 were robust to the various measurement factors. All variograms were modeled using Gaussian model that suggested the slow-changing spatial correlations for short distances of about several mm. This observation suggested that values of magnetic susceptibility and spatial correlations were not changing rapidly through the soil profile and were rather smooth. This observation could be explained by the characteristic soil processes that are rather stable in time and space. All variograms achieved distinctive sill at the distance of about 5 cm.

**Fig. 3:**
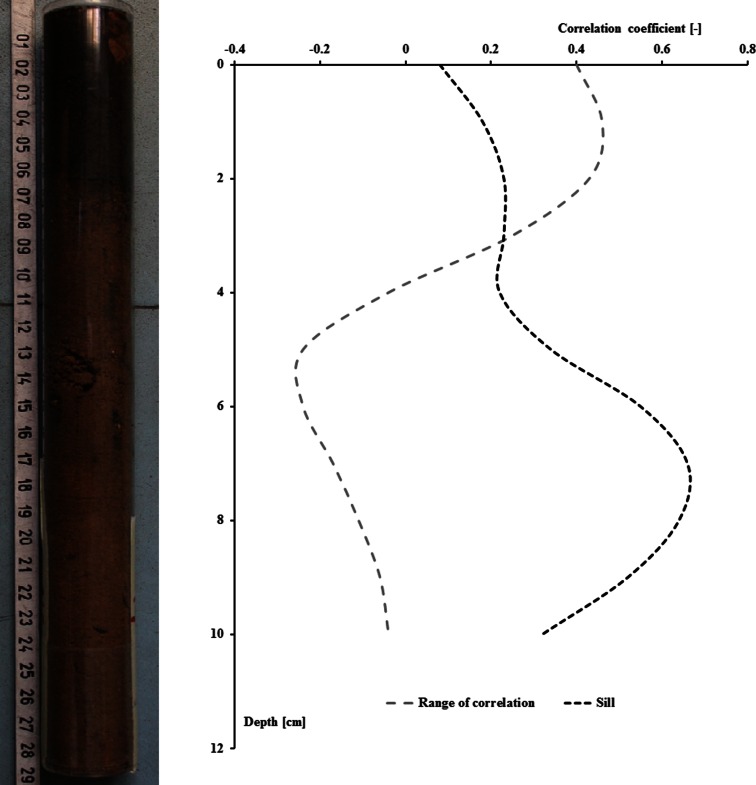
Plot of values of Pearson correlation coefficients between a range of correlation, a sill and soil magnetic susceptibility at depths ranging from 0 to 10 cm

The range of correlation of magnetic susceptibility in the oil profile was most significantly correlated with the values of magnetic susceptibility that were measured at first 3 cm of soil profile (Table 2). The strength of correlation with values of magnetic susceptibility measured deeper in the soil profile was decreasing (Fig. 3) and was statistically insignificant. As it was previously analyzed in this part of the soil profile, the most of the pollutants were usually accumulated. In consequence, the range of correlation was correlated only with this part of soil profile that was related to the anthropogenic pollution. Considering that the variogram and, in particular, the range of correlation takes into consideration the spatial characteristic of the analyzed phenomena, the range of correlation can be used as a some measure of the degree of pollutants’ dispersion in the soil profile.

**Table 2:** Pearson correlation coefficient between parameters of microscale spatial variability of κ value in soil profile and magnetic indicators

Magnetic indicator		Parameter of spatial variability	
		Range of correlation	Sill
Magnetic susceptibility at the depth of:	0 cm	**0.40**	0.08
	1 cm	**0.46**	0.18
	2 cm	**0.43**	0.23
	3 cm	0.25	0.23
	4 cm	−0.04	0.22
	5 cm	−0.24	**0.34**
	6 cm	−0.24	**0.55**
	7 cm	−0.17	**0.66**
	8 cm	−0.11	**0.64**
	9 cm	−0.06	**0.52**
	10 cm	−0.04	**0.32**
Area under the curve of magnetic susceptibility versus the depth in soil profile, calculated to depth of:	Background value	0.07	**0.63**
	6 cm	0.13	**0.33**
	8 cm	0.03	**0.54**
	10 cm	0.00	**0.68**
Maximum magnetic susceptibility		−0.05	**0.85**
Background value of magnetic susceptibility		−0.04	−0.17
Depth of maximum magnetic susceptibility		−0.08	0.07
Depth of background value of magnetic susceptibility		0.17	0.12
Average value of magnetic susceptibility		−0.01	**0.65**

Coefficients in bold were statistically significant (*α* = 0.05)

The range of correlation was rather poorly correlated with other magnetic measures such as the area under the curve of magnetic susceptibility versus a depth in the soil profile. Contrary, the sill of the variogram was significantly correlated with the area under the curve of magnetic susceptibility, and this may suggest that the sill of variogram can be directly related to the summary load of anthropogenic pollution of soil.

Furthermore, the sill of the variogram was also significantly correlated with the maximum magnetic susceptibility that was observed in the soil profile that was previously used as a measure of soil pollution. This confirms the observation of significant correlation between the sill and the area under the curve of magnetic susceptibility.

Additionally, the sill of the variogram was rather uncorrelated with the depths where maximum and background values of magnetic susceptibility were observed in soil profile. This may suggest that the sill of variogram, correlated to the summary load of anthropogenic pollution of soil, does not depend on the location of accumulated pollutants in the soil profile.

## Conclusions

The results showed that the range of correlation of magnetic susceptibility (κ) was significantly correlated with magnetic susceptibility measured at depths 1, 2, and 3 cm in the soil profile. It suggests that the range of correlation is a good measure of pollutants dispersion in the soil profile, especially in the upper soil layers where the most of anthropogenic pollutants are usually accumulated.

The sill of the variogram of magnetic susceptibility was found to be significantly correlated with the area under the curve of plot of magnetic susceptibility. The highest correlation was observed between the sill of variogram and the area under the curve of plot of magnetic susceptibility calculated to the depth of 10 cm and the depth of background value of magnetic susceptibility. Area calculated to these depths was good representation of the total magnetic load of anthropogenic pollution. Moreover, the sill of the variogram was uncorrelated with the depths where maximum and background values of magnetic susceptibility were observed in soil profile that suggests that the sill does not depend on the location of accumulated pollutants in the soil profile.

In summary, it can be concluded that the parameters of microscale spatial variability of magnetic susceptibility in the soil profile can be useful to estimate both the load and the degree of dispersion of pollutants in the soil profile.
